# Mental health problems in pregnancy link maternal history of childhood maltreatment to altered newborn amygdala connectivity

**DOI:** 10.21203/rs.3.rs-10092045/v1

**Published:** 2026-10-06

**Authors:** Vanessa Babineau, Seonjoo Lee, Zhijun Zhang, Zheshu Jiang, Elizabeth Werner, Jay Ginrich, Jonathan Posner, Bradley Peterson, Catherine Monk

**Affiliations:** Icahn School of Medicine at Mount Sinai; Duke University; University of Southern California

## Abstract

Childhood maltreatment affects 25% of the U.S. population and increases risk for psychopathology, including anxiety disorders, across generations. Before postnatal exposures, offspring with a mother who has a history of childhood maltreatment are born with greater functional connectivity in amygdala-prefrontal stress circuits. Similarly, maternal stress during pregnancy associates with both greater positive and greater inverse amygdala-prefrontal connectivity in infants in a sex-specific manner. Altered newborn amygdala-prefrontal circuits that support stress processing may reflect accelerated neural maturation. Prior studies on associations between maternal history of childhood maltreatment and newborn amygdala connectivity adjust for prenatal maternal stress yet have not examined whether these associations are mediated by prenatal maternal stress. Because history of childhood maltreatment strongly correlates with maternal mental health problems during pregnancy, including stress, depression, sleep problems, and pregnancy specific distress, we test whether these problems mediate the association between maternal history of childhood maltreatment and altered newborn amygdala-prefrontal connectivity. Participants (N = 59) report their history of childhood maltreatment and mental health symptoms in the 3rd trimester. We measure resting state functional connectivity using fMRI in infants (mean age = 3.15 ± 1.42 weeks). Prenatal maternal sleep problems mediate associations between maternal history of childhood maltreatment and infant’s amygdala connectivity in both sexes. Pregnancy specific distress mediates the associations in males. Overall, females show greater positive connectivity, whereas males show greater inverse connectivity; mediation findings are more consistent in males. Our study provides evidence that prenatal maternal mental health problems – particularly sleep problems and pregnancy specific distress – link maternal history of childhood maltreatment to newborn amygdala connectivity. These findings highlight modifiable prenatal factors for trauma-responsive intervention.

## INTRODUCTION

Childhood maltreatment affects approximately 25% of the U.S. population^1^ and increases risk for mental health disorders across generations.^2^ Maltreatment includes emotional, physical, and sexual abuse, as well as emotional and physical neglect.^3,4^ For example, one study reports that 40% of children with a mother who has a history of childhood maltreatment develop an anxiety disorder, compared to 24% of children without such exposure.^5^

Most established pathways of intergenerational trauma are through postnatal behavioral exposures that involve mechanisms such as maternal mental health problems and decreased parenting sensitivity,^6^ whereas biological pathways remain less well characterized.^2^ Maternal mental health and parenting behaviors represent key exposures that link a mother’s history of childhood maltreatment to outcomes in the next generation.^6,7^ A maternal history of childhood maltreatment increases risk for maternal mental health problems, as well as for reduced parenting sensitivity, emotional unavailability, hostility, and neglect.^6^ These factors, in turn, contribute to offspring emotion dysregulation, disorganized attachment, social-emotional difficulties, and psychopathology.^6,8^ A maternal history of childhood maltreatment also correlates with socioeconomic and health related risk factors – including lower income, lower educational attainment, and obesity – that impact fetal and infant development. ^6,9-11^

Prenatal programming provides a framework to investigate biological pathways of intergenerational transmission before birth. This framework, grounded in the Developmental Origins of Health and Disease (DOHaD) hypothesis, proposes that maternal characteristics during pregnancy shape fetal development and susceptibility to disease.^12,13^ The maternal-placental-fetal unit integrates stress-sensitive biological signals that influence fetal growth and brain development.^14^ A maternal history of childhood maltreatment associates with alterations in endocrine and immune function, including increased hypothalamic-pituitary-adrenal (HPA) axis reactivity, which may influence fetal stress regulation.^14^ These biological signals may alter fetal brain development and increase susceptibility to stress-related psychopathology.^14,15^

Emerging evidence links maternal history of childhood maltreatment to altered fetal and newborn amygdala-prefrontal functional connectivity, a network central to stress processing.^10,16^ In fetuses, maternal history of childhood maltreatment associates with greater amygdala connectivity with prefrontal and premotor regions.^16^ In newborns, maternal history of childhood maltreatment associates with increased newborn amygdala connectivity with the dorsal anterior cingulate cortex (ACC) and ventromedial prefrontal cortex (vmPFC) only after adjusting for prenatal maternal stress.^10^ Amygdala-prefrontal circuits support emotion regulation by shaping HPA-axis responses and autonomic reactivity to stressors.^17^ Alterations in amygdala circuitry associate with chronic stress and psychiatric disorders, including depression and anxiety in children and adults.^17-20^

Direct assessment of newborn brain connectivity allows identification of prenatal influences before postnatal environmental exposures. Prior work shows that maternal history of childhood maltreatment associates with increased amygdala-prefrontal connectivity in infants.^10^ In related studies, prenatal maternal depression associates with altered fetal neurobehavior and infant amygdala-prefrontal connectivity through indirect pathways,^21^ and prenatal maternal stress (i.e., a composite of depression, anxiety and stress) associates directly with amygdala-prefrontal connectivity.^22^ Additionally, elevated maternal cortisol levels in pregnancy associate with sex-specific patterns of altered newborn amygdala connectivity.^23^ Both increased positive and greater inverse connectivity within infant amygdala circuits reflect accelerated neural maturation of stress related networks,^10,24,25^ which may lead to early behavioral phenotypes such as negative affect.^26-28^

A maternal history of childhood maltreatment strongly associates with prenatal maternal mental health problems, including stress, depression, anxiety, sleep disturbances, and pregnancy specific distress.^6^ Because both maternal history childhood maltreatment and prenatal mental health associate with alterations in infant amygdala connectivity,^10,21^ these factors may operate along a shared biological pathway. Testing this pathway could refine indicators for early intervention. Prior work suggests that maternal history of childhood maltreatment and prenatal depression interact to influence newborn brain structure,^29^ indicating that maternal childhood maltreatment may influence infant brain development via altered maternal mood in pregnancy and associated gestational biology.

We investigate a novel pathway of intergenerational transmission for maternal history of childhood maltreatment through *in utero* exposure to maternal mental health problems. We test whether prenatal maternal mental health problems mediate the association between maternal history of childhood maltreatment and altered newborn amygdala connectivity, with regions of the PFC that contribute to stress processing. We also explore moderation by infant biological sex, with evidence to suggest sex-specific patterns in neural development.^30,31^ In a primarily exploratory hypothesis-generating analysis, we expect that greater prenatal maternal stress, depression, sleep problems, and pregnancy specific distress will mediate the relationship between maternal history of childhood maltreatment and accelerated maturation of newborn amygdala-PFC functional connectivity (to vmPFC, ACC, dorsal and medial superior frontal gyrus [dSFG and mSFG], precentral gyrus [PreCG]).

## MATERIALS AND METHODS

### Participants

Participants are 18 to 45 years of age with healthy singleton pregnancies, recruited through the Department of Obstetrics and Gynecology at Columbia University Medical Center. They are excluded if they acknowledge smoking or use of recreational drugs or are not fluent in English. Additional descriptions of the participants are published elsewhere.^21^ Participants provide informed consent; all procedures are approved by the New York State Psychiatric Institute Review Board. This is a secondary analysis of an existing dataset (NIMH P50MH090966-05).^21^

### Study procedures

During pregnancy at 34 to 37 weeks’ gestation, participants self-report history of childhood maltreatment, prenatal maternal mental health problems (i.e., stress, depression, sleep problems, pregnancy specific distress), and demographic information. Within weeks of birth, infants, while sleeping, undergo resting state functional magnetic resonance imaging (fMRI) to measure connectivity within amygdala-prefrontal circuits.

### Infant characteristics

Gestational age (GA) at birth and postmenstrual age (PMA) at scan session are determined by the last reported menstrual cycle. GA and birthweight are obtained from the medical chart.

### Maternal history of childhood maltreatment

Participants complete the self-reported Childhood Trauma Questionnaire (CTQ), a 28-item retrospective measure of childhood maltreatment, with five subscales including emotional abuse, physical abuse, sexual abuse, emotional neglect, and physical neglect.^32^ The total score is summed and averaged; higher scores indicate greater childhood maltreatment experiences.

### Prenatal maternal mental health

Participants complete four self-report measures of mental health during pregnancy, including the Perceived Stress Scale (PSS), a 10-item measure of perceived stress over the past month; the Center for Epidemiological Studies Depression Scale (CES-D), a 20-item measure of depressive symptoms over the past week; the Pittsburgh Sleep Quality Index (PSQI), a 19-item measure of sleep problems over the past month; and the Pregnancy Distress Questionnaire (PDQ), a 12-item measure of pregnancy specific distress (e.g., worries and concerns about pregnancy). For each measure, a total score is summed and averaged; on the PSS, CES-D, PSQI, and PDQ, higher scores indicate greater prenatal maternal mental health problems. The predetermined thresholds for depression and sleep problems are used for descriptive purposes and to test for potential covariance (i.e., CES-D ≥ 16 or PSQI ≥ 5, respectively).^33,34^

### Infant amygdala functional connectivity

#### MRI parameters

Functional imaging is acquired on a GE Signa 3T whole body scanner with an eight-channel head coil. Acquisition of a three-dimensional spoiled gradient recall image is followed by echoplanar images (TR = 2200 ms, TE = 30 ms, 90° flip angle, single excitation per image, slice thickness 3.5 mm, 19 × 19 cm FOV, 64 × 64 matrix, 24 slices) with an effective resolution of 3.75 × 3.75 × 3.5 mm and whole-brain coverage. Five runs of 102 volumes (3 min and 44.4 s) are obtained for each participant.

#### Head motion

Several steps are taken to limit the in influence of head motion. First, any fMRI run with a peak Framewise Displacement (FD) over 1 mm is excluded.^35^ Second, the number of fMRI runs across subjects is matched by selecting two of the five total runs with the least head motion (all participants have two usable runs except for two participants having only one). Infants with peak FD values > 0.5 mm are removed. Third, frames with excessive motion are censored. Additional head motion parameter are estimated with DVARS.^35^

### Functional connectivity image processing

For image processing, we use the NeoRS preprocessing pipeline.^36^ Briefly, NeoRS registers T2-weighted images to an age-specific stereotaxic template in Talairach space at 1 and 3 mm isotropic resolution using a single-step 12-DOF affine transformation without resampling. Skull stripping is performed in template space using FSL bet2,^37^ and segmentation is performed using the SPM-based MANTiS toolbox^38^ to generate eight tissue probability maps. Masks resampled to an isotropic resolution of 3 mm to match the functional image space for downstream confound regression. The blood-oxygen-level-dependent (BOLD) images undergo slice-timing correction using FSL slicetimer and head motion correction via rigid-body registration (6 degrees of freedom) of each volume to the first volume using FSL mcflirt with zero smoothing and sinc interpolation. BOLD images are registered to a 3 mm isotropic Washington University neonatal template using a single 12-DOF affine registration. Motion censoring and confound regression is performed with FD > 0.25 mm and 24 parameter motion regressors, mean white matter and CSF signals extracted from conservatively thresholded and eroded tissue probability masks, and a gray-matter-derived global signal. Any runs with more than 30% of outliers (FD > 0.25 mm) are excluded. Band-pass filtering is performed to retain BOLD fluctuations in the 0.01–0.1 Hz. Spatial smoothing is performed with a Gaussian kernel (full width at half maximum 6 mm). The denoised BOLD data are parcellated using the Automated Anatomical Labeling (AAL)^39^ atlas in template space, and regional mean time series are computed for each region of interest (ROI). The amygdala (left and right hemispheres) functional connectivity to prespecified ROIs (vmPFC, ACC, dSFG, mSFG, PreCG; left and right hemispheres) is generated using Pearson’s correlation coefficients.

### Statistical analyses

#### Path analysis and mediation

Path analysis and mediation testing are performed in R. We use multivariate general linear regression models with amygdala-prefrontal functional connectivity correlations as the dependent variable. We perform path analyses to explore the interrelationship between path (a) maternal history of childhood maltreatment to prenatal maternal mental health problems, path (b) prenatal maternal mental health problems to amygdala-prefrontal connectivity, path (c) maternal history of childhood maltreatment to amygdala-prefrontal connectivity without adjusting for prenatal maternal mental health problems, and path (c’) maternal history of childhood maltreatment to amygdala-prefrontal connectivity adjusting for prenatal maternal mental health problems. Average Causal Mediation Effect (ACME) models (i.e., prenatal maternal mental health problem as the mediator between maternal history of childhood maltreatment and amygdala-prefrontal connectivity) are tested with bias-corrected bootstrapping (10,000 iteration). All models are additionally stratified by infant biological sex at birth. P-values are not adjusted because this is primarily an exploratory hypothesis-generating analysis. In a sensitivity analysis, regression models are re-run removing infants exposed to selective serotonin reuptake inhibitors (SSRIs) in pregnancy.

#### Covariates selection

All models are adjusted a priori for infant PMA at scan and gravidity, and additional covariates considered based on the literature are retained if they are significantly associated with either the predicator (maternal history of childhood maltreatment) or outcome (amygdala-prefrontal connectivity). Additional covariates considered are infant biological sex and birthweight, and maternal age, pre-pregnancy body mass index (BMI), relationship status, self-reported race, self-reported ethnicity, household income, education, parity, and meeting the predetermined threshold for depression or sleep problems (i.e., CES-D ≥ 16 or PSQI ≥ 5, respectively).^33,34^

## RESULTS

### Descriptive characteristics

Detailed demographic and descriptive characteristics are in Table 1, and maternal history of childhood maltreatment and prenatal maternal mental health characteristics are in Table 2. Pregnant participants are 20 to 42 years of age (mean = 29.75 ± 5.56); 11.6% are taking SSRIs during pregnancy; most self-report as Black/African American (30.%), Other (40.7%), or White (25.4%) and as Hispanic/Latina (61%); most report a relationship status as living together (25.4) or married (45.8%); and most report a household income ≤ 50k per year (59.3%) or 51-100k per year (22.03%) (Table 1). During pregnancy, 33.9% of participants endorse a history of childhood maltreatment (any abuse and/or neglect), 13.8% meet the predetermined threshold for depression and 76.7% for sleep problems (Table 2). Functional MRI data are available for 61 infants; we remove two infants due to head motion (peak FD > 0.5 mm). Our final analysis includes N = 59 infants (61% biologically female) mean age = 3.15 ± 1.42 weeks at fMRI scan (see Table 1).

### Path analysis

The path analysis to support significant mediation outcomes is in Supplement Table S1. Path (a) demonstrates that greater maternal history of childhood maltreatment leads to significantly greater mental health problems in pregnancy (i.e., sleep problems and pregnancy specific distress) (*p*-value range .01 to < .001). Path (b) demonstrates a pattern where greater prenatal mental health problems (i.e., sleep problems and pregnancy specific distress) lead to alterations in newborn amygdala to prefrontal connectivity, with significant results in 50% of models (*p*-value range .04 to .01) and trends in the remaining 50% of models (*p*-value range .05 to .07). Path (c) and (c’) demonstrate that when we adjust for mental health problems in pregnancy (i.e., sleep problems and pregnancy specific distress), we observe significant associations between greater maternal history of childhood maltreatment and alterations in newborn amygdala to prefrontal connectivity in 43% of models (*p*-value range .03 to < .01). Finally, we observe the ACME where higher prenatal maternal sleep problems and pregnancy specific distress mediate associations between greater maternal history of childhood maltreatment and alterations in newborn amygdala to prefrontal connectivity (*p*-value range .04 to .01).

### Mediation by prenatal maternal mental health problems

A summary of significant mediation results is in Table 3. Higher prenatal maternal sleep problems mediate the association between greater maternal history of childhood maltreatment and alterations in newborn amygdala-prefrontal connectivity (to ACC, dSFG, mSFG, PreCG), specifically greater positive connectivity in females and greater inverse connectivity in males (Table 3). Higher maternal pregnancy specific distress mediates the association between greater maternal history of childhood maltreatment and greater newborn inverse amygdala-prefrontal connectivity (to vmPFC, dSFG, mSFG, PreCG) in males (Table 3; Fig. 1). Significant results are more consistent in males. There are no differences in lateralization. To illustrate the predominant pattern of mediation results, we focus on the mediation effect by higher prenatal maternal sleep problems (β = −0.80, [CI: 1.80, −0.10], p = .02) on the association between greater maternal history of childhood maltreatment and greater inverse newborn connectivity between the right amygdala (right hemisphere) to the left ACC (left hemisphere) in males (Table 3); these results are illustrated as a path analysis in Fig. 2 and as a scatter plot in Fig. 3.

### Sensitivity analysis

In a sensitivity analysis excluding n = 7 infants exposed to SSRIs in pregnancy, few models remain significant, yet the pattern of results remains similar (see Supplement Table S2). The models that remain significant are those in which higher prenatal maternal sleep problems mediate the association between maternal childhood maltreatment and greater inverse newborn amygdala to prefrontal connectivity (to ACC and PreCG) in males.

## DISCUSSION

Using resting state fMRI, we investigate whether maternal mental health problems in pregnancy, including stress, depression, sleep problems, and pregnancy specific distress, mediate the association between maternal history of childhood maltreatment and alterations in infant amygdala-prefrontal connectivity to regions that contribute to stress processing (i.e., vmPFV, dSFG, mSFG, PreCG). We find that prenatal maternal sleep problems mediate associations between maternal history of childhood maltreatment and alterations in newborn amygdala-PFC connectivity (to ACC, dSFG, mSFG, PreCG), with greater positive connectivity in females and greater inverse connectivity in males. Further, prenatal maternal pregnancy specific distress mediates associations between maternal history of childhood maltreatment and greater newborn inverse amygdala-PFC connectivity (to vmPFC, dSFG, mSFG, PreCG) in males. Associations are most consistent in males and indicate sex-specific patterns to *in utero* exposures.^40,41^ We provide evidence that maternal mental health problems in pregnancy mediate the association between maternal history of childhood maltreatment and altered newborn amygdala connectivity.

Increased positive and greater inverse functional connectivity in infants, between the amygdala and regions of the PFC that contribute to stress processing, are interpreted as accelerated neural maturation^10,21^ consistent with the stress acceleration hypothesis.^42^ Across species, exposure to early life stress and adversity can accelerate maturation of biological systems, including prioritization of a more mature or adult-like stress response system to more readily detect threat.^42^ Accelerated neural maturation can prepare the infant for a challenging postnatal environment, with superior cognitive and psychomotor skills in the first year of life; whether neural priming poses risk or advantage can depend on congruence between pre- and postnatal environments.^43^ Alterations in amygdala circuits can be at the cost of dysregulated stress and sex hormones, early puberty, anxiety disorders, and depression.^18-20,44-46^

Alterations in infant amygdala-prefrontal connectivity are associated with infant behavioral phenotypes that lead to increased risk for childhood mental health problems.^26-28^ Greater infant amygdala-cingulate and superior frontal gyrus connectivity are associated with greater negative affect (e.g., more crying) and lower positive affect (e.g., less smiling).^26^ Similarly, greater newborn amygdala to insula and vmPFC connectivity is associated to greater infant fear and sadness.^28^ Additionally, infant amygdala increased connectivity to the salience network, and decreased connectivity to the executive control network, mediates the effect of maternal postnatal depression and anxiety on reduced infant smiling.^27^ Alterations in newborn amygdala connectivity are associated with infant behavioral phenotypes that are indicators of risk for later psychopathology.

We find sex-specific patterns in newborn amygdala-prefrontal connectivity. Specifically, mediation by prenatal maternal sleep problems in both sexes and by pregnancy specific distress in males, of associations between maternal history of childhood maltreatment with greater positive connectivity in females and greater inverse connectivity in males between the amygdala to PFC. This is corroborated by a previous study in which elevated maternal cortisol in pregnancy is associated with sex-specific patterns in amygdala connectivity, with greater positive connectivity in females and greater inverse connectivity in males.^23^ We also find that males are more consistently affected than females. The sexually dimorphic nature of the placenta, where female placentas appear better able to adapt to prenatal insults, leaves males at increased vulnerability for accelerated neural maturation.^30,40,41,47^ Previous association studies between maternal history of childhood maltreatment or prenatal stress with alterations in fetal and newborn amygdala connectivity find no clear sex differences or do not test moderation by sex.^10,16,21,48^ In related work, sex-specific patterns in functional brain connectivity are confirmed in the fetus,^49^ including greater cross-region connections in females and within region connections in males.^50^ In a study on the development of whole brain functional connectivity within and between-networks in the first two years of life, males demonstrate faster interhemispheric integration of large-scale networks supporting executive function.^51^ Sex-specific patterns in amygdala-prefrontal functional connectivity in newborns exposed to prenatal maternal stress – with or without maternal history of childhood maltreatment – remain less well characterized. Our results of sex-specific patterns in newborn amygdala-prefrontal connectivity – including greater positive connectivity in females and greater inverse connectivity in males corroborated by previous work^23^ – call for longitudinal investigation of continuity and discontinuity in association with sex-specific behavioral phenotypes and psychopathology.

Our findings support a novel biological pathway of intergenerational transmission of maternal history of childhood maltreatment through maternal mood affecting altered newborn amygdala connectivity in the next generation. In our path analysis, greater maternal history of childhood maltreatment is associated with greater symptoms of prenatal maternal mental health problems, and greater symptoms of prenatal maternal mental health problems are associated with accelerated maturation of amygdala connectivity. Before mediation testing, we replicate findings from a previous study^10^ concluding that maternal history of childhood maltreatment is associated with accelerated maturation of infant amygdala to prefrontal connectivity when adjusting for mental health problems in pregnancy. We add to these findings that greater prenatal maternal sleep problems and pregnancy specific distress mediate the effect of maternal history of childhood maltreatment on accelerated maturation of newborn amygdala connectivity in the next generation. In related work, the influence of prenatal maternal depressive symptoms on newborn global white matter microstructure is found to be dependent of maternal history of childhood maltreatment.^29^

Prenatal maternal perceived stress and depression are not significant mediators in our study. Approximately 14% of our study meets the threshold for depression during pregnancy, compared to an estimated 17% prevalence in the general population and a range of 8 to 26% internationally.^52^ Perceived stress scores in our study are comparable to previous pregnancy studies.^10^ Individuals with a history of childhood maltreatment are more likely to live with chronic stress and depression;^6^ pregnancy specific distress and sleep problems in pregnancy might be less common experiences accompanied by unknown symptoms compared to familiar lifelong stress and depression. Further, pregnancy and transition to parenthood can activate traumatic memories and emotions from childhood and being parented, and related biological responses.^6,53^ The unfamiliar symptoms of pregnancy specific distress and sleep problems in pregnancy and unique emotionally activating nature of pregnancy and transition to parenthood might be experienced with heightened distress and associated biological responses, offering a potential explanation for altered offspring neurodevelopment under certain heightened distress conditions compared to more common lifelong stress.

In a sensitivity analysis, we remove infants exposed to SSRIs during pregnancy. Few models remain significant, yet the pattern of results is similar. The models that remain significant despite the loss of statical power indicate that prenatal maternal sleep problems mediate associations between maternal history of childhood maltreatment and greater newborn inverse amygdala connectivity to ACC and PreCG in males. These results further support the mediating role of prenatal maternal sleep problems for males in the context of maternal history of childhood maltreatment.

Strengths of this study are the longitudinal design from pregnancy to the first weeks of life and newborn brain imaging to determine biological pathways of intergenerational trauma and transmission of risk for psychopathology before postnatal influences. We provide evidence of mediation during pregnancy, specifically by pregnancy specific distress and sleep problems, indicating a window for prevention and early intervention. Among our limitations, our small sample size limits our power for mediation models. Further, p-values are not adjusted to correct for multiple comparisons because this is an exploratory hypothesis-generating study. Additionally, we rely on retrospective reports of childhood maltreatment that are critiqued to have a poor agreement with prospective measures of these experiences; maltreated children identified prospectively (e.g., hospital or court records) may have different developmental trajectories and pathways to mental illness in adulthood than adults who retrospectively report memories of childhood maltreatment.^54,55^ Finally, we do not have the opportunity to demonstrate associations between changes in amygdala connectivity with infant behavior; this presents a line of inquiry for future investigation.

Our findings support a novel biological pathway of intergenerational trauma through mental health in pregnancy linking maternal history of childhood maltreatment experiences to altered newborn amygdala connectivity, with sex-specific patterns. These findings highlight modifiable prenatal factors for trauma-responsive interventions with a two-generation impact. Identifying a history of childhood maltreatment in pregnancy creates a window of opportunity to monitor prenatal maternal mental health and provide prevention and early intervention to support mother-infant pairs and healthy brain and behavior development across generations.

## Figures and Tables

**Figure 1:**
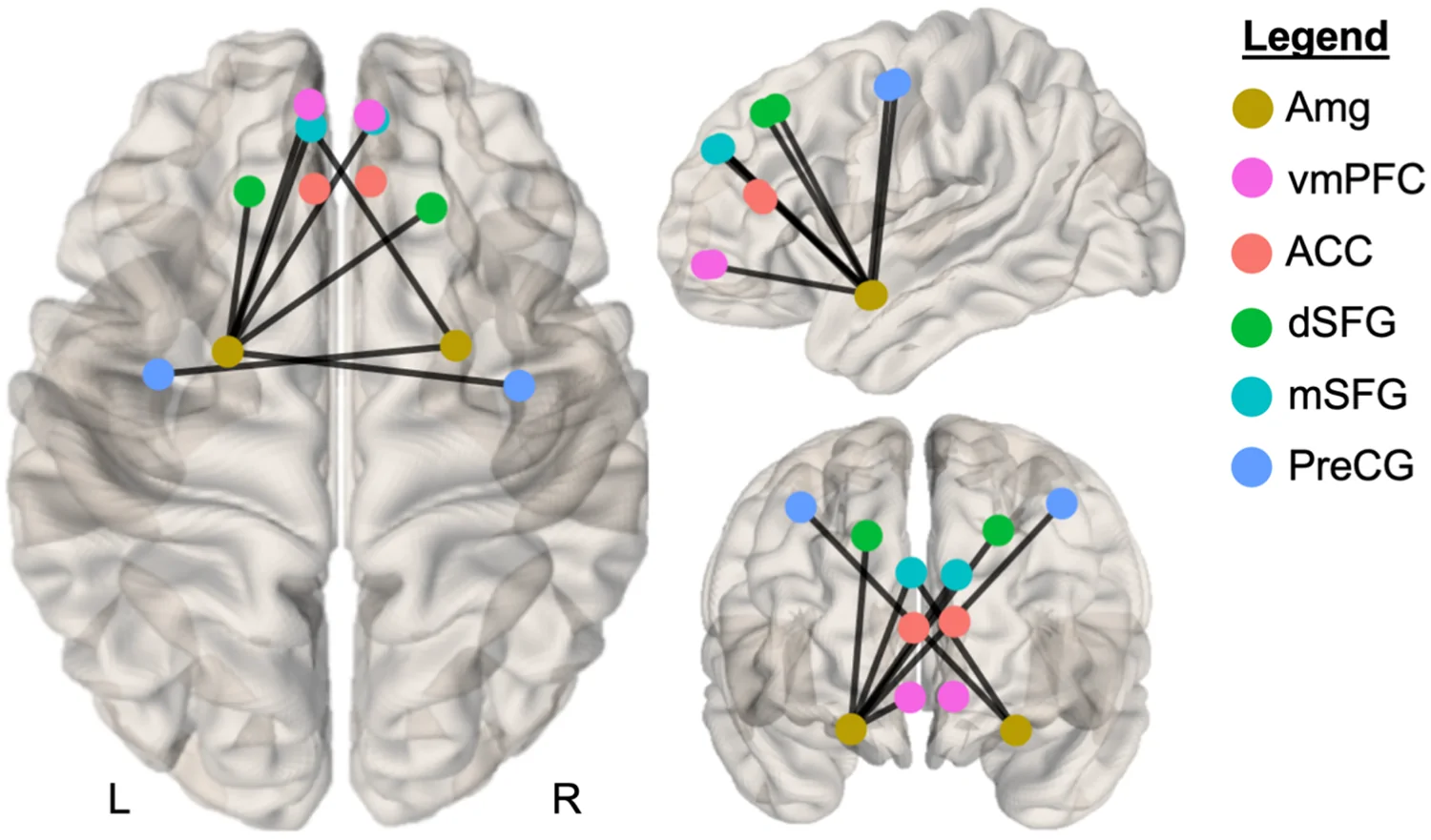
Illustration of greater inverse amygdala-prefrontal connectivity in males, a result of greater prenatal maternal sleep problems (measured on the Pittsburgh Sleep Quality Index [PSQI]) mediating the effect of maternal history of childhood maltreatment (measured on the Childhood Trauma Questionnaire [CTQ]). Specifically, amygdala (Amg) left hemisphere (L) to ventromedial prefrontal cortex (vmPFC)(L); Amg(L) to precentral gyrus (PreCG) right hemisphere (R); Amg(R) to PreCG(L); Amg(L) to dorsal superior frontal gyrus (dSFG)(L); Amg(L) to dSFG(R); Amg(L) to medial superior frontal gyrus (mSFG)(L); Amg(L) to mSFG(R); Amg(R) to mSFG(L).

**Figure 2:**
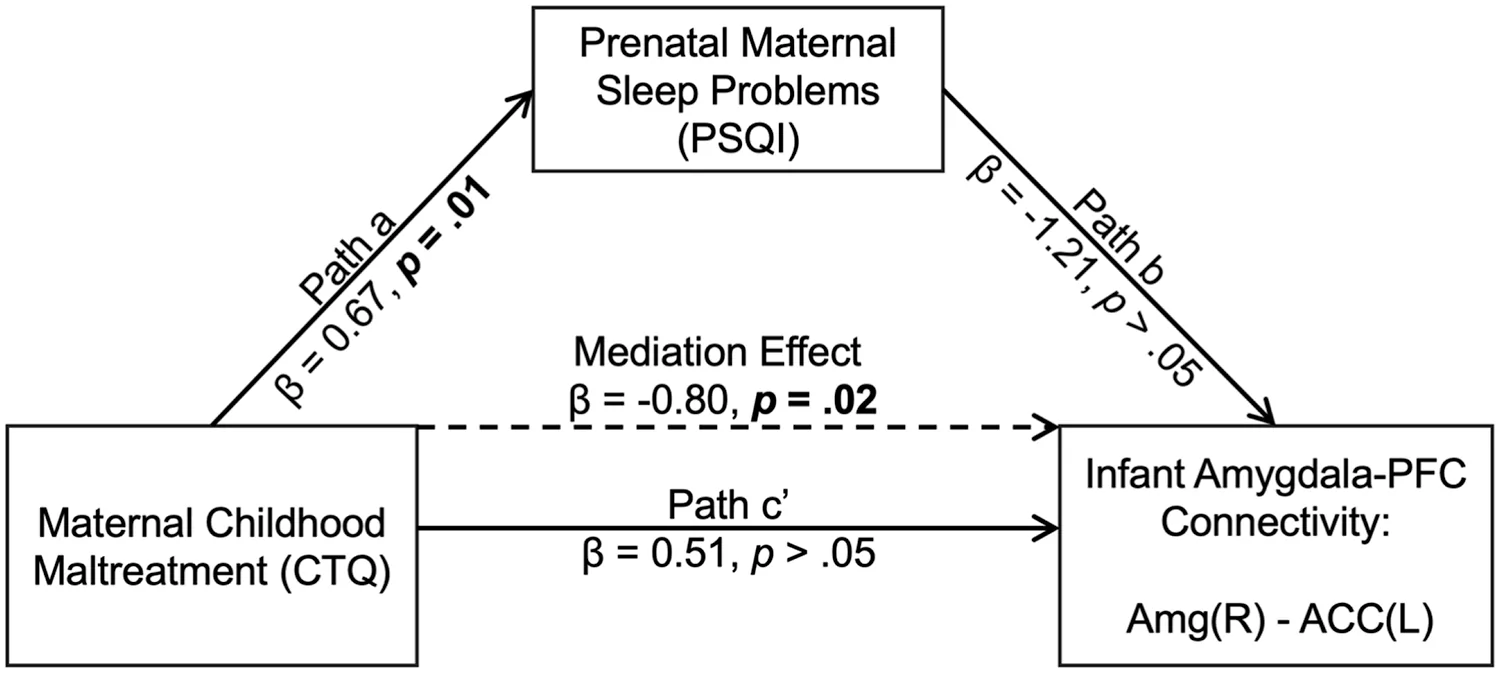
The path analysis illustrates one example of the mediation effects summarized in Table 3. The path analysis suggests that higher prenatal maternal sleep problems (measured on the Pittsburgh Sleep Quality Index [PSQI]) mediate the association between greater maternal history of childhood maltreatment (measured on the Childhood Trauma Questionnaire [CTQ]) and altered amygdala-prefrontal cortex (PFC) connectivity in males; specifically greater inverse amygdala (Amg) right hemisphere (R) to anterior cingulate cortex left hemisphere (ACC[L]). In the first linear regression (Path a), we find that the CTQ is a significant predictor of the PSQI (β = 0.67, *p* = .01). In the second linear regression (Path b), we find no association between the PSQI and male infant amygdala-PFC connectivity (Amg[R]-ACC[L]) (β = −1.21, *p* = .05). In the third linear regression (Path c’), we find no association between the CTQ and male infant amygdala-PFC connectivity when adjusting for the PSQI (Amg[R]-ACC[L]) (β = 0.51, *p* = .24). In mediation testing, the PSQI mediates the effect of the CTQ on male infant amygdala-PFC connectivity (Amg[R]-ACC[L]) (β = −0.80, *p*= .02). All models are adjusted for infant postmenstrual age at scan, maternal relationship status, ethnicity, and gravidity.

**Figure 3:**
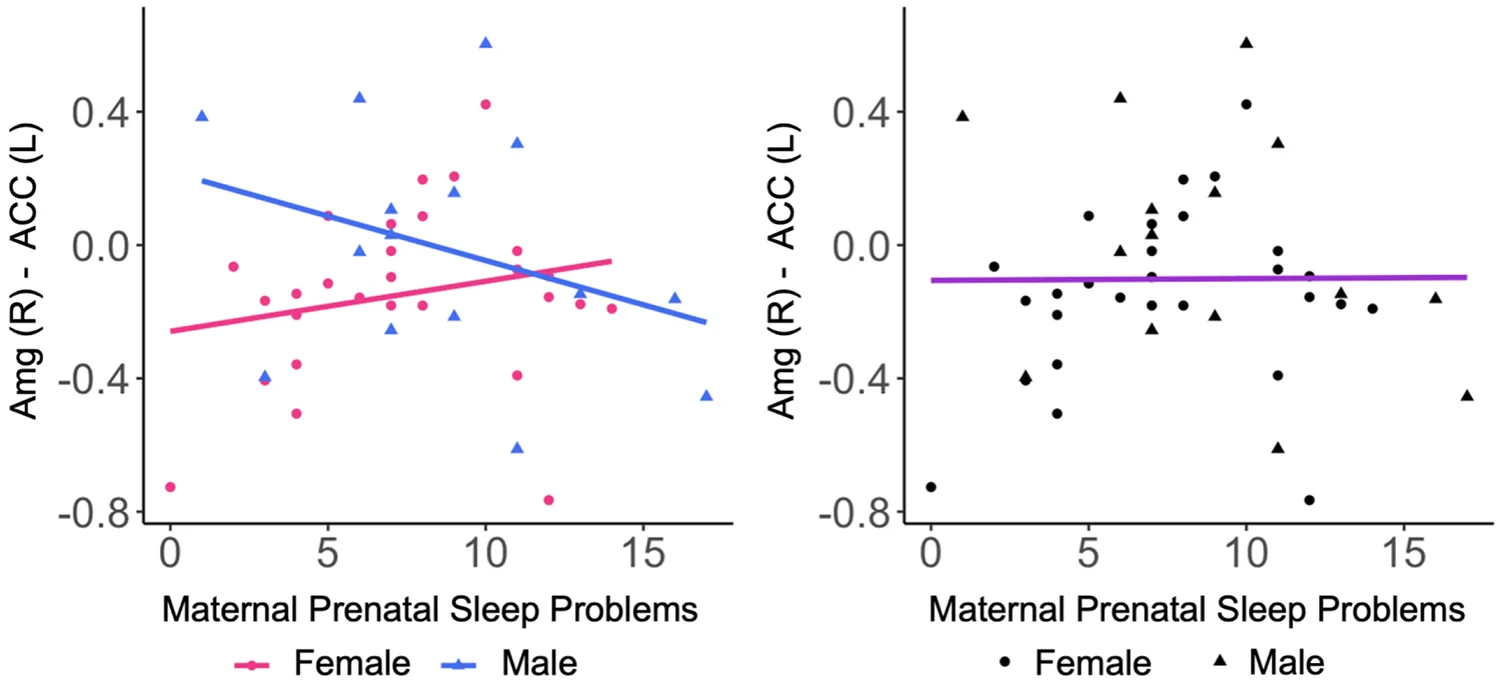
The scatterplot on the left demonstrates that greater prenatal maternal sleep problems (x-axis) (measured on the Pittsburgh Sleep Quality Index [PSQI]) mediate the association between maternal history of childhood maltreatment (measured on the Childhood Trauma Questionnaire [CTQ]) and amygdala-prefrontal cortex (PFC) connectivity (y-axis) in males (blue); specifically greater inverse amygdala (Amg) right hemisphere (R) to anterior cingulate cortex left hemisphere (ACC[L]) (Mediation Effect, β = −0.80, *p* = .02). The scatterplot on the right demonstrates no mediation effect in the overall group. All models are adjusted for infant postmenstrual age at scan, maternal relationship status, ethnicity, and gravidity.

**Table 1:** Demographics for N = 59 Pairs

Gestational age at birth (weeks)	Mean (SD), Range	*n* (%)
	39.24 (1.05), 35.29–41	
Postmenstrual age at scan (weeks)	42.39 (1.50), 38.29 – 45	
Gestational age at scan (weeks)	3.15 (1.42), 1.00–7.29	
Infant biological sex - female		36 (61.0%)
Birthweight (grams)	3,233.47 (431.04), 2275–4110	
Maternal age (years)	29.75 (5.56), 20.7–42.83	
Maternal BMI	30.98 (6.93), 20.13–57.16	
SSRIs during pregnancy		7 (11.86%)
Gravidity	2.07 (1.93), 0–9	
Parity	0.86 (0.99), 0–4	
Relationship status		
Living together	-	15 (25.4%)
Married	-	27 (45.8%)
Not living together	-	9 (15.2%)
Single	-	8 (13.6%)
Self-reported maternal race		
American Indian/Alaskan	-	1 (1.7%)
Biracial	-	1 (1.7%)
Black/African American	-	18 (30.5%)
Other	-	24 (40.7%)
White	-	15 (25.4%)
Self-reported maternal ethnicity		
Hispanic/Latina – Yes	-	36 (61.0%)
Household Income		
$0–15,000	-	16 (27.1%)
$16,000–25,000	-	3 (5.1%)
$26,000–50,000	-	16 (27.1%)
$51,000–100,000	-	13 (22.03%)
$101,000–250,000	-	10 (16.9%)
> $250,000	-	1 (1.7%)
Maternal education (years)	14.85 (3.25), 8–23	

**Note:** SD = Standard deviation; BMI = body mass index; SSRIs = selective serotonin reuptake inhibitors (antidepressant medication).

**Table 2:** Maternal History of Childhood Maltreatment and Prenatal Maternal Mental Health Characteristics

Maternal history of childhood maltreatment (CTQ)	Mean (SD)	Range	*n*(%) Threshold
		
Emotional abuse	8.31 (4.60)	5–24	7 (11.9)
Emotional neglect	9.03 (4.83)	5–24	7 (11.9)
Physical abuse	7.58 (4.69)	5–25	11 (18.6)
Sexual abuse	6.14 (3.36)	4–25	9 (15.3)
Physical neglect	5.95 (2.04)	5–18	2 (3.4)
Total CTQ score	37 (14.95)	24–95	n/a
Any abuse or neglect			20 (33.9)
Prenatal maternal mental health			
Perceived stress (PSS)	20.43 (7.32)	5–42.5	n/a
Depression (CES-D)	10.54 (8.8)	1–50	8 (13.8)
Sleep problems (PSQI)	7.91 (3.96)	0–17	33 (76.7)
Pregnancy distress (PDQ)	4.09 (3.07)	0–13	n/a

**Note:** SD = standard deviation; n/a = not applicable due to no pre-determined threshold; higher scores indicate greater symptoms or behaviors. Maternal history of childhood maltreatment is measured on the Childhood Maltreatment Questionnaire (CTQ) with predetermined thresholds for emotional abuse ≥ 13, physical abuse ≥ 10, sexual abuse ≥ 8, emotional neglect ≥ 15, and physical neglect ≥ 10. Perceived stress is measured on the Perceived Stress Scale (PSS), prenatal maternal depression is measured on the Center for Epidemiologic Studies Depression Scale (CES-D) with predetermined threshold ≥ 16, prenatal maternal sleep problems are measured on the Pittsburgh Sleep Quality Index (PSQI) with predetermined threshold ≥ 5, and maternal pregnancy distress is measured on the Pregnancy Distress Questionnaire (PDQ).

**Table 3:** Summary of Mediation Effects: Prenatal Maternal Mental Health Maternal Problems Mediate Associations between Maternal History of Childhood Maltreatment and Newborn Amygdala Functional Connectivity

Mediator / Outcome	Overall Sample (N = 59)			Males (n = 23)			Females (n = 36)		
	β	*p*-value	95% CI	β	*p*-value	95% CI	β	*p*-value	95% CI
Prenatal maternal sleep problems									
Amg(R)-ACC(L)*	-	-	-	−0.80	.02	−1.80, −0.10	-	-	-
Amg(L)-PreCG(L)	-	-	-	−0.57	.04	−1.34, −0.02	-	-	-
Amg(R)-dSFG(L)	0.31	.03	0.02, 0.67	-	-	-	0.40	.04	0.01, 0.97
Amg(R)-dSFG(R)	-	-	-	-	-	-	0.37	.04	0.01, 0.9
Amg(L)-mSFG(L)	-	-	-	−0.51	.04	−1.21, −0.02	-	-	-
Maternal pregnancy specific distress									
Amg(L)-vmPFC(L)	-	-	-	−0.38	.04	−0.88,−0.01	-	-	-
Amg(L)-PreCG(R)	-	-	-	−0.39	.04	−0.90, −0.01	-	-	-
Amg(R)-PreCG(L)	-	-	-	−0.39	.01	−0.85, −0.05	-	-	-
Amg(L)-dSFG(L)	-	-	-	−0.39	.02	−0.86, −0.04	-	-	-
Amg(L)-dSFG(R)	-	-	-	−0.32	.01	−0.70, −0.04	-	-	-
Amg(L)-mSFG(L)	-	-	-	−0.42	.01	−0.88, −0.08	-	-	-
Amg(L)-mSFG(R)	-	-	-	−0.38	.01	−0.79, −0.08	-	-	-
Amg(R)-mSFG(L)	-	-	-	−0.28	.04	−0.65, −0.01	-	-	-

**Note:** All models are adjusted for infant postmenstrual age at scan, maternal relationship status, ethnicity, and gravidity. Prenatal maternal sleep problems are measured on the Pittsburgh Sleep Quality Index (PSQI), and maternal pregnancy distress is measured on the Pregnancy Distress Questionnaire (PDQ). CI = confidence interval; Amg = amygdala; (L) = left hemisphere; (R) = right hemisphere; ACC = anterior cingulate cortex; PreCG = precentral gyrus; dSFG = dorsal superior frontal gyrus; mSFG = medial superior frontal gyrus; vmPFC = ventromedial prefrontal cortex.

* Demonstrated in Figures 2 and 3.
